# Self-enhancement in moral hypocrisy: Moral superiority and moral identity are about better appearances

**DOI:** 10.1371/journal.pone.0219382

**Published:** 2019-07-05

**Authors:** Mengchen Dong, Jan-Willem van Prooijen, Paul A. M. van Lange

**Affiliations:** 1 Beijing Key Laboratory of Applied Experimental Psychology, National Demonstration Center for Experimental Psychology Education (Beijing Normal University), Faculty of Psychology, Beijing Normal Univerisity, Beijing, P. R. China; 2 Department of Experimental and Applied Psychology, Faculty of Behavioral and Movement Sciences, VU Amsterdam, Amsterdam, the Netherlands; Middlesex University, UNITED KINGDOM

## Abstract

People often consider themselves as more moral than average others (i.e., moral superiority) and present themselves as more moral than they actually are (i.e., moral hypocrisy). We examined whether feelings of moral superiority—as a manifestation of self-enhancement motives—motivates people’s hypocritical behavior, that is, their discrepant moral performances in public versus private settings. In three studies (total *N* = 1,151), participants distributed two tasks (one favorable and one unfavorable) between themselves and an anonymous partner, with the option of using an ostensibly fair randomizer (e.g., a self-prepared coin). We found that when experiencing feelings of moral superiority (vs. non-superiority), people, especially those who highly identified with moral values (Studies 1 and 2), were less likely to directly give themselves the favorable task, but they were not less likely to cheat in private after using the randomizer (Studies 1 to 3). Both self-enhancement motives and moral identity have implications for hypocritical behavior, by motivating public moral appearances but not private moral integrity.

## Introduction

In everyday life, we may witness a fair amount of moral behaviors. People often do not take the last piece of pizza, they seldom cut in line when waiting, and help an elderly person cross the street. Do people engage in moral actions because they are genuinely concerned about the well-being of others, or merely because they want to *appear* moral to others? These questions touch upon a classic issue in the social and behavioral sciences—especially in public situations—that moral behavior can be motivated by either self-focused or other-focused motivations [1, 2].

These questions are related to the phenomenon of moral hypocrisy, which is to “appear moral, yet, if possible, avoid the cost of actually being moral” [3]. Put differently, people may often present themselves as moral in public while privately reaping the benefits of selfishness [4]. Although moral hypocrisy is prevalent in social life and ingrained in human behavior, little has been done to illuminate the motivational roots of hypocritical behavior.

The current research aims to test a self-enhancement account for why people often display moral hypocrisy. Self-enhancement is a fundamental human motive to maintain and strengthen a positive view of the self [5–7]. One of the most prominent manifestations of self-enhancement is that people often consider themselves as superior to others [8–10], especially in moral domains [11, 12]. We propose that people display moral hypocrisy because public moral appearance contributes to such self-enhancement motives. People’s moral behavior in response to stimuli that activate self-enhancement motives therefore should be more pronounced in public than private settings. Based on this rationale, in three studies we manipulated participants’ feelings of moral superiority, and examined its effects on both public and private moral choices.

### Moral hypocrisy: Public vs. private behavior

The motivation behind moral behavior can be multifaceted. People behave morally not only because they want to do good to others but also because they want to look good in the eyes of others, or feel good about themselves. People oftentimes present themselves in a moral manner, not only to make a favorable impression on others (i.e., impression management), but also to maintain a positive self-view (i.e., self-enhancement; [13]).

To manage interpersonal impression, people seem motivated to appear moral in public but not necessarily being so in private. For instance, people behave more morally in public (vs. private) settings where their behavior is under surveillance [14, 15], or where their individual commitment is visible (vs. invisible) to all group members [16]. Moral behavior in public effectively serves pragmatic goals of, for example, improving or sustaining their positive reputations [17] and earning them higher social status [16, 18]. Also, people act morally in front of others to conform with salient social norms [19, 20], thus avoiding third-party punishment incurred by norm transgressions [21].

Although much evidence has accumulated on the impression management account of why people behave more morally in public than in private [22], little is known about the role of self-enhancement motives in moral choices. People regulate their public (more so than private) moral performances, not only to gain reputational or material benefits but also to internalize them as support of their positive self-regard [4, 13, 23]. When given opportunities to cheat while keeping their reputation unharmed, therefore, people deceive moderately but not excessively to sustain a moral self-image [4]. People are willing to spend money, to avoid decision-making situations that may lead to others’ suffering [24, 25] and subsequent personal distress [1]. Complementing impression management motives, here we investigate how self-enhancement motives would influence the discrepancy between public and private morality, conceptualized as a unique behavioral pattern: moral hypocrisy.

Batson and colleagues [3, 26, 27] first conceptualized moral hypocrisy as public displays of morality combined with private selfishness. In a typical experiment [26], participants were asked to decide how to assign two tasks, one with and one without positive consequence (i.e., winning raffle tickets), between themselves and an anonymous partner. They were given three choices, namely to (1) assign the positive task to themselves, (2) assign the positive task to their partner, or (3) flip a coin to decide. Although approximately half of the participants chose the seemingly fair procedure of a coin flip, about 90% (9 out of 10 participants) of them subsequently “won” the coin toss and assigned the positive task to themselves. This proportion was significantly higher than the expected 50% chance if they had flipped the coin fairly. In other words, many people tried to appear moral in public (by choosing to flip a coin) but did not flip the coin fairly in private.

In sum, moral behavior differs in public versus private settings, and the discrepancies between public versus private moral behavior within the same domain often constitute moral hypocrisy [3, 27, 28]. In the present research, we investigate moral hypocrisy by separating its components of public from private moral behavior, and propose that self-enhancement motives contribute to moral hypocrisy by increasing public but not private moral behavior.

### Self-enhancement, moral superiority, and moral hypocrisy

Self-enhancement is a strong motivation underlying human cognition and behavior, and aims to promote or sustain a positive self-perception [5–7]. One of the most prominent manifestations of self-enhancement is that people often evaluate themselves as superior to their average peers [8, 29, 30]. Feelings of self-superiority are particularly salient in the moral (vs. intelligence, [11, 12]) domain, and may emerge irrespective of people’s actual position relative to others [31, 32]. When feelings of self-superiority are threatened by comparative others, people may even derogate others’ morality to glorify themselves [33].

How do feelings of moral superiority influence moral behavior? Previous research does not provide consistent evidence. Some studies suggest no effect of moral superiority on moral behavior. Measured feelings of moral superiority were unrelated to either trust in others in a trust game, or trustworthiness in a dictator game [34]. However, other studies suggest a positive effect of moral superiority on subsequent moral behavior. After gaining superior moral feelings by criticizing another person, people behaved more morally in subsequent tasks [35]. Even general feelings of self-superiority, induced by a feedback of superior (vs. inferior) task performance to average others, increased people’s willingness to help in a subsequent unrelated task [36]. Here we argue that the social context in which moral behavior takes place makes a difference; specifically, feelings of moral superiority promote public but not private moral behavior. We presume that feelings of moral superiority highlight self-enhancement motives, which stimulates public moral appearances but not private moral integrity [37]. Moral hypocrisy serves *interpersonal* impression management processes; here we mainly address its *intrapersonal* significance in boosting one’s positive self-concept.

Moral behavior can better signal one’s superior moral standing if performed in public rather than in private, not only *to others* (as impression management process; [16, 18, 38]) but also *to oneself* (as self-enhancement process). Sociometer theory [39, 40] suggests that self-esteem (in a similar vein with self-enhancement) serves an interpersonal monitoring function, by incorporating social appraisals into self-esteem feelings. The motivation to self-enhance thus fuels more public moral behavior, inherently characterized by exposure to others’ positive appraisals. To self-enhance, people also avoid immorality more in public than in private because it is more difficult to publicly as opposed to privately justify transgressions [41, 42]. Supporting this proposition, people exaggerated their superiority over others after private failures while expressing more egalitarian evaluations of self and others after exposure to a public failure [41]. Moral superiority feelings and self-enhancement processes hence are less susceptible to private than public immorality. Consistently, in Batson and colleagues’ moral hypocrisy studies [3, 26], although many people privately cheated to assign themselves the positive task after choosing to flip a coin, the public choice of flipping a coin still made them feel morally superior to those who directly gave themselves the positive task. Based on the above rationale, we reason that moral superiority should enhance moral hypocrisy by increasing public but not private moral behavior.

### The current research

In three studies, we investigated the influence of moral superiority on moral hypocrisy to reveal the self-enhancement mechanism underlying discrepancies in public versus private moral behavior. We adopted a revised online version of Batson and colleagues’ [3, 26, 27] coin-flipping paradigm as indicator of people’s hypocritical behavior. In the current paradigm, online participants were asked to distribute two arithmetic tasks differing in length. Consistent with previous studies [3, 26, 27, 33], participants should generally find the tasks tedious from our descriptions (i.e., a task to “examine how time length and fatigue would influence people’s performance on basic mental arithmetic”), and prefer the shorter to the longer task. As indicator of public moral behavior, they first chose from (1) assigning the shorter task to themselves, (2) assigning the shorter task to their partner, or (3) using a 50/50 randomizer to decide (i.e., a self-prepared coin in Studies 1 and 3 and a computerized randomizer in Study 2). If they indicated to use the randomizer, they were subsequently asked for the final assignment of tasks following the randomizer’s result. Given the private nature of cheating in using the randomizer (especially when flipping a self-prepared coin; Studies 1 and 3), we consider people’s second choice as the indicator of their private moral behavior.

We conducted the experiments online and recruited participants from crowdsourcing websites (i.e., CrowdFlower and Prolific Academic, participants of which are more diverse and naïve than of Amazon Mechanical Turk [43]), to both guarantee the anonymity of private moral behavior and the reputational context of public moral behavior. Online participants of these platforms seek to become highly reputable workers (as indicated by their approval rates), to gain more paid opportunities in the future [44].

Furthermore, in all three studies, we measured moral identity as a continuous independent variable to explore its potential role in the relationship between moral superiority and moral hypocrisy. Moral identity is an additional source of moral motivation [45, 46]. However, little research has addressed how moral identity influenced moral hypocrisy, especially the comparative effects of moral identity on public versus private moral behavior. Although previous studies suggest that a strong moral identity suppresses immoral behavior and promotes moral integrity [47, 48], we presume that moral identity also reflects sensitivity to social approvals, such that measured moral identity should positively predict moral behavior in public but less so in private. Supporting this proposition, Krettenauer and Casey [49] reasoned that moral identity is not only concerned with integrity to moral beliefs and values, but also with demonstrating morality to others. While confirming the positive association between moral identity and moral behavior, a recent meta-analysis of 111 studies [50] showed that moral identity yielded stronger effect sizes as a predictor of self-reported, rather than behavioral or field third-party observations of, moral behavior. Both self-reported (vs. observed) and public (vs. private) exhibition of moral behaviors can get inflated by reputational concerns and self-presentation bias [51–53], and thus be more responsive to moral identity measures.

Below we examine the effects of moral superiority and moral identity on moral hypocritical behavior, conceptualized as public vs. private moral behavior. Studies 1 to 3 followed the same procedure: Participants first completed a moral identity measure [48], then were primed with their feelings of moral superiority (vs. non-superiority; with a recalling-and-writing task in Study 1 and bogus personality test feedbacks in Studies 2 and 3), and eventually completed the distribution task measuring their hypocritical behavior.

## Study 1

In this first study, we used a recalling paradigm to induce feelings of moral superiority to others and examined its effects on moral hypocrisy. We included two comparison conditions: A moral inferiority condition, and a controlled recalling condition. We expect that people in the moral superiority condition would be more likely to make moral choices in public (e.g., flipping a coin) than in the moral inferiority and control conditions. After choosing to flip the coin, however, we do not expect them to cheat less in private.

### Method

#### Participants and ethics statement

Two hundred and sixty-four American participants completed our online experiment on CrowdFlower. After excluding 46 participants according to one well established attention check question—“Please choose the first option indicating strongly disagree” [54]—on a 7-point scale (1 = *strongly disagree* to 7 = *strongly agree*), we had a final sample of 218 participants (94 male; *M*_age_ = 36.2 years, *SD* = 11.3). We have obtained the required permits and ethical approvals required of foreign researchers. The studies reported in this manuscript were approved by our faculty’s ethical review board (VCWE; NO. 2017–044). Across the three studies, participants provided electronic consent prior to taking the online survey. Upon completion of the studies, participants were debriefed online.

### Procedure

Participants first completed basic demographics and the moral identity measure [48]. They were then randomly assigned to one of the three moral standing conditions: the moral superiority recalling, the moral inferiority recalling, and the controlled recalling group. After the moral standing manipulation, all participants completed an online task distribution procedure assessing their hypocritical behavior.

We first presented the pictorial measure of social mindfulness [55] as distraction task and then asked participants to complete the Self-Importance of Moral Identity Questionnaire [48]. With internalization and symbolization as two subscales of the moral identity measure, we only employed the internalization subscale in the current research given its higher validity in predicting moral outcomes [50]. Participants were asked to first read nine prototypical adjectives describing a moral person (i.e., caring, compassionate, fair, friendly, generous, helpful, hardworking, honest, and kind) and to visualize the kind of person who possesses these characteristics. We then presented five items measuring moral identity (e.g., “It would make me feel good to have these characteristics”, ranging from 1 = *strongly disagree* to 7 = *strongly agree*). After reversely coding the third and fourth items (as in [48]), the five items were averaged into a reliable moral identity scale (*α* = 0.81).

To manipulate moral standing, participants read a cover story that we were interested in collecting some scenarios that “helped them understand their status relative to others” from “their comparison with the person(s) in a situation where they were present at the same time”. They were asked to recall a recent episode after reading three different versions of instructions as below. In the moral superiority condition (*n* = 70), participants read “…Someone else or some other people did something morally indecent (e.g., cheating, littering, smoking in public, etc.). Instead, you behaved well, or you thought you would definitely do better than them in the same situation. You may feel that their behavior reflected their lack of some moral characters (e.g., honesty, kindness, generosity, compassion, etc.) and that you were superior to them in these moral aspects.” Correspondingly, in the moral inferiority condition (*n* = 69), participants were asked to recall an experience in which someone else or some other people “did something morally decent (e.g., helping, donating, volunteering, etc.)” and make participants feel “inferior to them in some moral aspects”. In a controlled recalling condition (*n* = 79), we asked participants to describe a typical day in their life. One question—“How do you appraise your morality relative to average others?”—was set as manipulation check for the moral standing manipulation (rated on a 7-point scale from 1 = *far above average* to 7 = *far below average*; reverse-coded).

We then utilized an online coin-flipping paradigm [3, 26, 27] to examine people’s public and private moral behavior. Specifically, we told participants that there would be two versions of arithmetical tasks of equivalent difficulty but different lengths (Task 1 takes 5 min and Task 2 takes 20 min). To guarantee equal numbers of people in both tasks, they would be online paired with an anonymous participant and one of them would be randomly selected to decide how to assign these two tasks. In fact, all participants would be instructed to play the role of “Distributor”. They were given three choices to (1) directly choose Task 1, (2) directly choose Task 2, or (3) use a self-prepared coin to decide (Choice 1). When choosing the third option in Choice 1, they were then asked to flip a self-prepared coin and indicate their coin-flipping result (Choice 2: choosing Task 1 or Task 2). At the end of the experiment, participants were debriefed online.

### Results

#### Manipulation check

We first examined the effectiveness of moral standing priming with the manipulation check question. Participants in the three conditions differed significantly in their subjective feelings of moral standing, *F* (2, 215) = 4.11, *p* = .02, η_p_^2^ = .04. As intended, pairwise comparisons (LSD) showed that participants in the superiority condition (*M* = 5.21, *SD* = 1.03) perceived themselves as more moral as compared to both the control (*M* = 4.78, *SD* = 1.22, *p* = .02, *d* = 0.38) and inferiority (*M* = 4.75, *SD* = 0.91, *p* = .01, *d* = 0.47) conditions; however, participants did not differ in their feelings of moral standing in the inferiority vs. control condition, *p* = .86. Thus, recalled experiences of moral superiority to specific target(s) caused superior moral feelings, while experiences of moral inferiority to specific target(s) did not evoke generalized feelings of moral inferiority.

#### Public moral appearance

Participants’ choices in Studies 1 through 3 were shown in Table 1. Different from previous studies (e.g., [3, 22, 26, 27]), which solely targeted the choice of the randomizer as the public component of moral hypocrisy, we aggregated Task 2 (the longer task) and the randomizer as the manifestation of public moral behavior (i.e., Task 1 = 0 and Task 2/randomizer = 1) across all three studies. Aggregating these choice options fits the current research question, which investigated public and private displays of morality relatively independently. In the public choice specifically, both the longer Task 2 and the randomizer can serve self-enhancement motives by showing public morality, as compared the shorter Task 2 representing the selfish choice. We mean-centered the moral identity score (*M* = 5.93, *SD* = 1.05) and specified two orthogonal contrasts for the moral standing conditions: V1 represented the effect of moral superiority versus the other two conditions (superiority = 2, control/inferiority = -1), and V2 represented the effect of moral inferiority versus the control condition (superiority = 0, control = -1, inferiority = 1). The main effects (V1, V2, moral identity) and the two-way interactions were entered into the binary logistic regression equation in two steps (see S1 Table).

**Table 1:** Overview of the participants’ choices in Studies 1, 2 and 3.

		Frequency and percentage		
		Task 1 (5 min)	Task 2 (20 min)	Randomizer
**Study 1**	Choice 1 (218 people)	98 (45%)	19 (8.7%)	101 (46.3%)
	Choice 2 (101 people)	69 (68.3%)	32 (31.7%)	_
**Study 2**	Choice 1 (326 people)	72 (22.1%)	18 (5.5%)	236 (72.4%)
	Choice 2 (233 people)	205 (88%)	28 (12%)	_
**Study 3**	Choice 1 (607 people)	321 (52.9%)	44 (7.2%)	242 (39.9%)
	Choice 2 (242 people)	168 (69.4%)	74 (30.6%)	_

The interaction between V1 and moral identity had a significant effect on Choice 1 (*B* = 0.20, *SE* = 0.10, Wald’s *χ*^2^ = 4.06, *Exp(B)* = 1.22, *p* = .04). Although the simple slopes were non-significant, superior moral standing, compared to non-superior moral standings (i.e., moral inferior and control conditions), tended to make those of high moral identity (+1 *SD*) less likely to choose selfishly in public (*B* = 0.22, *SE* = 0.14, Wald’s *χ*^2^ = 2.29, *Exp(B)* = 1.24, *p* = .13) but to make those of low moral identity (-1 *SD*) more likely to choose selfishly in public (*B* = -0.20, *SE* = 0.15, Wald’s *χ*^2^ = 1.85, *Exp(B)* = 0.82, *p* = .17). Furthermore, in the morally superior condition (V1 = 2), people’s moral identity positively predicted their public moral choices (*B* = 0.59, *SE* = 0.24, Wald’s *χ*^2^ = 5.79, *Exp(B)* = 1.79, *p* = .02); however, in non-superior conditions (V1 = -1), the relationship between moral identity and public moral choices was non-significant (*B* = 0.02, *SE* = 0.16, Wald’s *χ*^2^ = 0.01, *Exp(B)* = 1.02, *p* = .91). None of other predictors had a significant effect on the public Choice 1 (*p*s > .13).

#### Private moral behavior

Although we could not establish whether participants indeed cheated after (allegedly) flipping the self-prepared coin, their final choice of assigning the shorter task to themselves revealed a higher likelihood of cheating when the general proportion of choosing the shorter task was significantly higher than the expected 50% chance level. Indeed, after indicating to have flipped a coin, 68.3% of participants in Study 1 chose the shorter task (coded as 0) instead of the longer task (coded as 1) and this proportion largely deviated from the chance level of 50%, *t* (100) = -3.94, *p* < .001, which indicated that an estimated 18.3% of the participants cheated in their private task assignments. To examine the effects of moral standing and moral identity on the potential cheating behavior, we again conducted a binary logistic regression concerning participants’ Choice 2 after choosing to flip a coin. None of the predictors had a significant influence on participants’ choices between the shorter and the longer task after flipping the self-prepared coin, that is, their probability of private cheating (*p*s > .19, see S2 Table).

### Discussion

Study 1 revealed that the combination of high moral superiority and moral identity influenced people’s moral behaviors in public but not in private circumstances. When people experienced moral superiority (vs. inferiority or non-moral feelings), moral identity only predicted more moral choices in public but not in private; people of high moral identity were equally likely to cheat in private as compared to low moral identity others. The findings suggest that moral superiority and moral identity predicted people’s desire to appear moral instead of actually being moral—a pattern that is consistent with the notion of moral hypocrisy.

## Study 2

Study 2 aimed to replicate these findings with two main changes. First, we employed a more direct manipulation of moral superiority in Study 2. Participants were presented with personal morality scores relative to most others after a bogus personality survey, such that they compared their morality with a broader population in Study 2 rather than concrete target(s) in Study 1. Study 1 did not find a difference between the moral inferior and control conditions; we speculate that it is difficult to induce feelings of moral inferiority in research participants. We therefore excluded the moral inferiority condition and focused on the effect of moral superiority, relative to feelings of moral equivalence, on hypocritical behavior in the next two studies. Second, we modified the public moral choice of self-prepared coin to a computerized randomizer. In Study 1, we interpreted people’s private Choice 2 of the shorter task as the indicator of cheating, but could not confirm which specific participants cheated. By employing a computerized randomizer instructing all participants to choose the longer task, we could tell with certainty that those who chose the shorter task after using the randomizer cheated.

### Method

#### Participants

Four hundred and thirty American participants completed our online survey via CrowdFlower. After ruling out 104 participants according to a similar attention check question as in Study 1—“Please choose the last option indicating strongly agree”—on a 7-point scale (1 = *strongly disagree* to 7 = *strongly agree*), we had a final sample of 326 participants (134 male, *M*_age_ = 36.1 years, *SD* = 12.4).

#### Procedure

After basic demographic questions, participant completed the moral identity measure as part of a bogus personality survey. We then presented bogus feedback to induce subjective feelings of moral superiority, after which participants continued with the distribution task.

We first told participants that they were enrolled in the development of a national personality survey and would be presented with sets of questions that were randomly selected from our question pool. In return, we would provide them with a brief analysis based on our current database. Participants then completed 9 items from the Portrait Value Questionnaire (PVQ, [56]) as distraction task and the moral identity measure (α = 0.84) as in Study 1. When participants completed all the so-called test questions, we randomly presented one out of two feedbacks to each participant. In the moral superiority condition (*n* = 155), participants saw an online page indicating that they “beat 89% participants on their morality scoring” and their morality level “is superior to most American people”. A corresponding paragraph of description was presented below: “Based on our analysis, we presume that your daily performance on morality is extraordinarily good. You can perfectly manage your moral attitudes and behavior and behave almost as perfectly as a moral exemplar. You regulate your moral performance much better than most other people do.” In the moral equivalent control condition (*n* = 171), the feedback differently indicated that the subject “beat 51% participants” and was morally “equivalent to most American people”. We then described their performance of morality as “not bad” and their regulation of moral performance as “as well as most other American people do”. Two follow-up questions were then presented as manipulation check: (1) “How well do you think the analysis describes you?” (rated on a 5-point scale ranging from 1 = *describe me extremely well* to 5 = *does not describe me*) and (2) “How do you appraise your morality relative to average others?” (rated on a 7-point scale ranging from 1 = *far above the average* to 7 = *far below the average*). Both of the items were reverse-coded for comprehension.

Following the personality test feedback, we then presented the online coin-flipping paradigm as an unrelated cognitive task. Study 2 replaced the choice “using a self-prepared coin” with “a computerized randomizer”. To make participants fully aware of the opportunity to cheat in Choice 2, we explained that after using the randomizer, a sentence indicating the task to which they are assigned would first be presented, and then they had to start their assigned task manually. When participants chose the computerized randomizer, they were always assigned to Task 2 (i.e., the 20-min task). As such, anyone who started Task 1 (i.e., the 5-min task) after choosing the randomizer cheated.

### Results

#### Manipulation check

The induction of moral superiority feelings with bogus test feedbacks worked as intended. Compared to the moral equivalence group (*M* = 3.23, *SD* = 1.01), people who received superior moral feedback felt that the feedback described them more appropriately (*M* = 3.74, *SD* = 0.81), *t* (324) = 4.91, *p* < .001, *d* = 0.56. Though participants in the superior (*M* = 5.33, *SD* = 1.02) and equivalent (*M* = 4.90, *SD* = 1.02) conditions consistently rated their moral level above others, participants in the superior group evaluated their morality more positively, *t* (324) = 3.80, *p* < .001, *d* = 0.42.

#### Public moral appearance

We included (1) moral standing conditions (superiority = 1 and equivalence = 0), mean-centered moral identity score (*M* = 6.08, *SD* = 0.96), and (2) their interaction in two steps of a binary regression analysis to examine their effects on the public moral Choice 1 (see S1 Table). Identical to Study 1, the interaction between moral superiority and moral identity was significant (*B* = 1.12, *SE* = 0.31, Wald’s *χ*^2^ = 13.51, *Exp(B)* = 3.07, *p* < .001). Among participants with high moral identity (+1 *SD*), feelings of moral superiority (vs. equivalence) increased the likelihood of choosing the randomizer or the longer task directly (*B* = 1.17, *SE* = 0.43, Wald’s *χ*^2^ = 7.56, *Exp(B)* = 3.22, *p* = .006). Among participants with low moral identity (-1 *SD*), however, moral superiority increased the likelihood of assigning themselves to the shorter task directly (*B* = -0.98, *SE* = 0.39, Wald’s *χ*^2^ = 6.52, *Exp(B)* = 0.37, *p* = .01). Interpreted differently, when feeling morally superior, people of high (vs. low) moral identity were more likely to behave morally in public (*B* = 0.88, *SE* = 0.24, Wald’s *χ*^2^ = 13.57, *Exp(B)* = 2.41, *p* < .001); however, this effect became non-significant when people felt morally equivalent to others (*B* = -0.24, *SE* = 0.19, Wald’s *χ*^2^ = 1.62, *Exp(B)* = 0.79, *p* = .20).

#### Private moral behavior

More than half (63.7%) of the participants behaved hypocritically by first choosing the fair procedure of randomizer (72.4%) and then cheated to give themselves the shorter Task 1 after being assigned to complete Task 2 (88%; see Table 1). We then conducted the two-step binary logistic regression analysis for private cheating behavior (Choice 2). We found that the main effect of moral identity predicted higher probability of private honesty, that is, choosing Task 2 as instructed by the randomizer (*B* = 0.43, *SE* = 0.19, Wald’s *χ*^2^ = 5.14, *Exp(B)* = 1.53, *p* = .02). However, neither the effect of moral superiority (*B* = -0.11, *SE* = 0.42, Wald’s *χ*^2^ = 0.07, *Exp(B)* = 0.89, *p* = .79) nor the interaction between moral superiority and moral identity was significant (*B* < -0.01, *SE* = 0.42, Wald’s *χ*^2^ < 0.001, *Exp(B)* > 0.99, *p* > .99). In sum, feelings of moral superiority predicted high moral identifiers’ choice of appearing moral but not their private moral behavior afterwards.

### Discussion

Study 2 replicated the joint effects of moral superiority and moral identity on public moral appearance but not on private cheating behavior. By employing a computerized randomizer with a fixed undesirable result, we confirmed that among participants who valued morality as their central identity, feelings of moral superiority (vs. equivalence) led them less likely to appear immoral but not less likely to cheat in private (i.e., choosing the shorter task after being assigned to the longer one). Consistent with Study 1, the effects of moral superiority on moral appearance (i.e., Choice 1) were moderated by individual differences in moral identity. Compared to people in the moral equivalence condition, those in the morally superior condition were more likely to appear moral only when they considered morality as central to their identity. In contrast, when people valued morality less as part of their self-concept, their feelings of moral superiority licensed them to publicly behave in self-serving ways (see for a review, [57]).

To identify private cheating behavior, the computerized randomizer may have compromised participants’ feelings of full anonymity. In a related manner, we found in Study 2 that moral identity predicted a decreased likelihood to cheat in private after choosing a randomizer. It is possible that this effect emerged because in Study 2 participants did not perceive the randomizer’s result as fully confidential. They might be concerned, for instance, that cheating behavior would be visible to others (e.g., the experimenters). Moreover, as compared to the self-prepared coin, the computerized randomizer merely informed participants of the (ostensibly) randomized result of the longer Task 2, which may induce ambiguity in participants’ perception of the randomization process.

## Study 3

The computerized randomizer may jeopardize participants’ feelings of anonymity and beliefs in the authenticity of randomization. To address these potential limitations, we again used the choice of a “self-prepared coin” in Study 3. Furthermore, Study 3 served as a final confirmatory study for our line of reasoning: We therefore targeted a larger sample size in Study 3 than in the previous studies, and preregistered the study on the Open Science Framework before implementation. All the materials can be accessed at https://osf.io/4br39/?view_only=b8b2ec86c6034897a0c0945e85a003d9.

### Method

#### Participants

We aimed *N* = 600 participants in our final sample. We recruited 710 American participants on the crowdsourcing website Prolific Academic, given that we would also use attention check questions to select valid participants for further analyses. Specifically, three predetermined check questions were employed. First, as in Studies 1 and 2, the statement “Please choose the last option indicating strongly agree” was presented amongst the moral identity measures. Then two comprehension check questions were presented to examine whether participants carefully read the instructions for assigning the two tasks: (1) “How is the time length of TASK 1 relative to TASK 2?” and (2) “How is the pay of TASK 1 relative to TASK 2?” In the instructions, we presented that Task 1 takes 5 min and Task 2 takes 20 min, and both of these tasks would be equally paid with 80 cents bonus after participants’ completion. So the targeted answers to these two questions should be “shorter” and “the same” respectively. Accordingly, we excluded 103 participants who failed any of these three questions and included the remaining 607 people in the analyses (304 male, *M*_age_ = 32.5 years, *SD* = 10.8).

#### Procedure

Study 3 used a similar procedure as in Study 2. After the moral identity measure (α = 0.80) and a pictorial measure of social mindfulness as distractor task, we experimentally induced moral superiority (*n* = 303) vs. moral equivalence (*n* = 304) with bogus personality test feedbacks and then asked participants to distribute two arithmetical tasks of different lengths between themselves and an anonymous partner.

We made some minor changes to the instructions as compared to Study 2. First, we modified the moral feedback, to describe a range of moral scores neutrally (e.g., “Your morality score is higher than 87% to 93% of previous participants”) instead of a specific number implying interpersonal competition in Study 2 (e.g., “You beat 89% of participants on the morality scoring”). Second, we changed the choice of “computerized randomizer” in Study 2’s task distribution procedure back to the option of “self-prepared coin” (cf. Study 1) to guarantee participants’ anonymity of their private moral behavior.

### Results

#### Manipulation check

We adopted the same two manipulation check questions as in Study 2. As intended, participants who received the moral superiority feedback (*M* = 3.31, *SD* = 1.03) evaluated the feedback more positively than those who received the moral equivalence feedback (*M* = 3.03, *SD* = 0.92), *t* (605) = 3.55, *p* < .001, *d* = 0.29. Moreover, as compared to the equivalent group (*M* = 4.96, *SD* = 1.04), participants in the moral superiority condition considered themselves as more moral than average others (*M* = 5.13, *SD* = 1.12), *t* (605) = 1.92, *p* = .06, *d* = 0.15.

#### Public moral appearance

As in Studies 1 and 2, we conducted binary logistic regression analyses, including moral superiority priming (superiority = 1 and equivalence = 0), moral identity, *M* = 6.21, *SD* = 0.80, and their two-way interaction as predictors, to examine their effects on Choice 1 and Choice 2 respectively. For Choice 1, the main effect of moral superiority (*B* = 0.34, *SE* = 0.16, Wald’s *χ*^2^ = 4.38, *Exp(B)* = 1.41, *p* = .04) and moral identity (*B* = 0.29, *SE* = 0.11, Wald’s *χ*^2^ = 7.22, *Exp(B)* = 1.33, *p* = .01) were both significant, while not for the interaction (*B* = -0.07, *SE* = 0.21, Wald’s *χ*^2^ = 0.10, *Exp(B)* = 0.94, *p* = .75). Participants behaved more morally in public (i.e., choosing Task 2 or randomizer) when they felt morally superior (51.2%) rather than equivalent to others (43.1%), and when they had stronger moral identity.

#### Private moral behavior

Similar to Study 1, 69.4% of the participants who chose to flip a self-prepared coin eventually indicated the shorter task for themselves, of which the proportion was significantly higher than the expected 50% chance level if participants flipped the coin fairly, *t* (241) = 6.54, *p* < .001. For Choice 2, however, none of the superior moral feedback (*B* = 0.02, *SE* = 0.28, Wald’s *χ*^2^ = 0.004, *Exp(B)* = 1.02, *p* = .95), moral identity (*B* = 0.12, *SE* = 0.19, Wald’s *χ*^2^ = 0.35, *Exp(B)* = 1.12, *p* = .55), or their interaction (*B* = -0.11, *SE* = 0.39, Wald’s *χ*^2^ = 0.08, *Exp(B)* = 0.90, *p* = .78) predicted participants’ choice between Task 1 and Task 2 after flipping the self-prepared coin, namely, the probability of cheating in the private setting.

### Discussion

Study 3 again revealed the effects of moral superiority and moral identity on public, but not private, moral behavior. At the same time, while the effect of moral superiority on moral appearance was moderated by moral identity in previous two studies, in Study 3 we found independent significant main effects of both moral superiority and moral identity. We will further address this discrepancy in the General Discussion. Here in Study 3 we found that people who felt morally superior (vs. equivalent) to average others and valued morality more (vs. less) as their central identity were both more likely to appear moral in public yet were equally likely to cheat in private. Moreover, we again confirmed the null effect of moral identity on private cheating behavior as in Study 1, such that in fully anonymous situations, people with high (vs. low) moral identity were equally likely to cheat.

## General discussion

People make different moral choices in public versus private settings. Batson and colleagues [3, 26, 27] conducted a series of landmark studies to empirically reveal people’s prevalent discrepancies between their public moral appearance and private moral behavior, that is, moral hypocrisy. People have this strong tendency to present themselves as more moral than they actually are in various domains of social life; however, previous studies rarely addressed why moral hypocrisy prevails in human behavior. In the current research, we propose that moral superiority, as a predominant manifestation of self-enhancement motives, influences public and private moral behavior differently and thus can illuminate the pervasiveness of moral hypocrisy. Our findings in three studies supported the above proposition.

Across three independent studies, we employed different methods to induce feelings of moral superiority, and consistently found that moral superiority increased public moral behavior (moderated by moral identity in Studies 1 and 2) but not private moral behavior. People generated superior moral feelings from both episodic comparisons with specific target(s) (Study 1) and short descriptions about their relative moral standing to average others (Studies 2 and 3). The moral superiority manipulations did not drastically alter people’s subjective self-evaluations (yielding relatively small effect sizes on the manipulation check, *d*s< 0.50), which can be partially attributed to (1) the valence of the manipulation, and (2) the high baseline level of moral self-evaluations. Self-enhancement stimulated by positive messages generally yields weaker effects than self-protection stimulated by threatening feedback [58, 59]. Moral superiority represents a strong belief that many people hold, and is therefore difficult to manipulate [11, 12] in a very substantial manner. Indeed, even when reminded of their moral equivalence (Studies 2 and 3) or inferiority (Study 1), people felt somewhat morally superior to average others (*p*s < .001, as compared to the midpoint 4 = equal to average on a 7-point scale). Nonetheless, people are highly sensitive to self-defining social appraisals [59]. The small difference of situational superior moral feelings may therefore have led to tangible behavioral changes in public settings in our studies.

Moreover, we found an overall effect of moral identity on public but not private moral behavior. Although many previous studies established that moral identity strengthens moral behavior and curbs immoral behavior [47, 60, 61], to our best knowledge, this is the first empirical research to reveal that moral identity is sensitive to the reputational context of moral behavior. Higher moral identity predicted more public moral choices (when people experienced moral superiority in Studies 1 and 2) but not necessarily genuine moral concerns. This is also revealed in the discrepant findings of private moral behavior. Moral identity failed to predict less cheating behavior in completely private and non-reputational contexts (Studies 1 and 3), while when the private opportunity of cheating became arguably traceable (at least by the experimenters), people of high moral identity were less likely to cheat (Study 2). Therefore, people’s self-reported importance of moral identity may reflect its importance in self-presentation.

The three studies provided support for the positive effects of moral superiority and moral identity on public (but not private) moral behavior, but yielded somewhat divergent results concerning how the effects took place. While we found in Study 3 that both moral superiority and moral identity independently increased the likelihood of public moral behavior, in Studies 1 and 2, the effects of moral superiority on public morality were moderated by individual differences in the extent to which people valued morality as their central identity (i.e., moral identity). When experiencing superior moral feelings, only people with stronger (vs. weaker) moral identity were more likely to enact public moral behavior. The latter findings are consistent with studies on the moral consistency vs. moral licensing effects, which suggest that an antecedent moral action prompts high moral identifiers to behave in accordance with their moral values but liberates low moral identifiers to do the opposite [57, 62, 63]. Both superior (vs. non-superior) social comparison with others and antecedent moral (vs. immoral) behavior can influence subsequent moral behavior by inducing positive moral feelings about the self.

The divergent findings on the effect of moral superiority on public moral behavior may be partially attributed to heterogeneity in moral identity in our samples. We conducted Welch’s *t*-tests to compare moral identity scores across the three studies with unequal sample sizes, and found that the average moral identity was substantially higher among participants in Study 3 (*M* = 6.21, *SD* = 0.80), as compared to participants in Studies 1 (*M* = 5.21, *SD* = 1.03, *t* (345) = 12.86, *p* < .001) and 2 (*M* = 6.08, *SD* = 0.96, *t* (615) = 1.99, *p* < .05). In other words, it is possible that the main effect of moral superiority in Study 3 essentially captured high moral identifiers’ more public moral choices, which is consistent with the interaction effect in Studies 1 and 2 showing that moral superiority strengthened public moral choices among people with high moral identity. Future research may further illuminate how moral superiority and moral identity influence public moral appearances among more diverse samples, and the potential overlap of moral social comparison and antecedent moral behavior as motivators of subsequent moral behavior.

### Limitations and future directions

The current research examined moral superiority as integral to social comparison with others, while perception of the self and others can be two independent components of self-superior feelings. People can self-enhance by exaggerating their own morality, but also, by emphasizing others’ immorality [33]. Future research may tease these two components apart and examine their respective contributions to the self-enhancement process of moral hypocrisy.

By employing an additional fair procedure of the randomizer, we investigated people’s hypocritical motives underlying their public moral behavior by examining their subsequent private choices. However, this paradigm is restricted in revealing the independent effect of moral superiority feelings on private behavior. Participants’ public choice of the randomizer was always set prior to their private moral choices, leading to possible selection or carry-over effects. For example, Kristofferson, White, and Peloza [64] showed that the public token support for a prosocial cause (e.g., signing a petition or not) would induce positive moral feelings and thus derogate subsequent contributions to the same cause (e.g., donating money or volunteering time). To provide more solid evidence for the distinct effects of moral superiority on public vs. private moral behavior, future studies may examine public vs. private behaviors in independent conditions.

Moreover, only participants choosing the randomizer necessarily were included in the private behavior condition, making it difficult to directly compare the moral superiority effects on behaviors performed in public vs. private settings. Furthermore, the null effect of moral superiority on private moral integrity may be due to the reduced sample size (and hence reduced statistical power) as compared to the public behavior condition. When examining the effect sizes, however, it turns out that in Studies 1 and 2 (among participants high in moral identity, i.e., + 1 *SD*), moral superiority increased the likelihood of public moral behavior (odd ratio = 1.24 and 3.22 respectively). As reported previously, on private moral behavior the effects of moral superiority were non-significant, but if anything, the effect sizes suggested a *lower* likelihood of private moral behavior in the moral superiority conditions (odd ratio = 0.72 and 0.89 respectively). Likewise, in Study 3 the effect size revealed a higher likelihood of public moral behavior (odd ratio = 1.41) in the morally superior versus equivalent moral group, while suggesting no differences in the likelihood of private moral behavior across conditions (odd ratio = 1.02). Although the three studies differed in sample size and experimental manipulation, the effect sizes consistently suggested that moral superiority feelings increased the likelihood of public but not private moral behavior.

## Conclusion

In three studies we found empirical relationships among moral superiority, moral identity, and moral hypocrisy. Stronger feelings of moral superiority caused more public choices of moral appearance, especially for those who valued morality as their central identity, but not more private choices of moral integrity. The patterns we observed are in line with the concept of moral hypocrisy introduced by Batson and colleagues [3, 26, 27], and suggest that seemingly moral behavior is often motivated by desires for positive self-views and the underlying self-enhancement motives rather than by moral motives, such as the desire to be impartial, fair, or considerate. One might speculate about the ultimate motives, such as the desire to remain part of a supporting group, or maintaining cooperative relationships with others. It is interesting that moral appearance seems easily activated, thus leading to moral hypocrisy. It also shows that reality is not always what it seems to be, and one of the next challenges is to illuminate the different psychological paths that lead to moral hypocrisy and to moral integrity.

## Supporting information

S1 Table Results of binary regression analyses for Choice 1 in Studies 1, 2 and 3. (DOCX)

S2 Table Results of binary logistic regression for Choice 2 in Study 1. (DOCX)

S1 Datasets Data and syntax of Studies 1, 2 and 3. (ZIP)
