# Supplementary material for: Self-enhancement in moral hypocrisy: Moral superiority and moral identity are about better appearances
Source: PLoS One. 2019 Jul 5;14(7):e0219382. doi: 10.1371/journal.pone.0219382 (PMC6611614; doi:10.1371/journal.pone.0219382)
Supplement: S1 Table — (DOCX) [file pone.0219382.s001.docx]

**S1 Table. Results of binary regression analyses for Choice 1 in Studies 1, 2 and 3.**

|  | | | | | 95% *CI* for *Exp(B)* | |
| --- | --- | --- | --- | --- | --- | --- |
|  | | *B(SE)* | Wald’s $\chi^{2}$ | *Exp(B)* | Lower | Higher |
| Study 1 | Constant | 0.20(0.14) | 2.21 | 1.22 |  |  |
|  | V1 | 0.01(0.10) | 0.02 | 1.01 | 0.84 | 1.23 |
|  | V2 | -0.07(0.17) | 0.19 | 0.93 | 0.67 | 1.29 |
|  | Moral identity | 0.20(0.13) | 2.26 | 1.22 | 0.94 | 1.57 |
|  | V1$\times$Moral identity | 0.20*(0.10) | 4.06 | 1.22 | 1.01 | 1.47 |
|  | V2$\times$Moral identity | 0.10(0.16) | 0.40 | 1.11 | 0.80 | 1.53 |
| Study 2 | Constant | 1.26(0.13) | 89.16 | 3.53 |  |  |
|  | Moral superiority | -0.01(0.27) | <0.01 | >0.99 | 0.59 | 1.69 |
|  | Moral identity | 0.17(0.13) | 1.55 | 1.18 | .91 | 1.53 |
|  | V1$\times$Moral identity | 1.12***(0.31) | 13.51 | 3.07 | 1.69 | 5.59 |
| Study 3 | Constant | -0.12(0.08) | 2.02 | 0.89 |  |  |
|  | Moral superiority | 0.34*(0.16) | 4.38 | 1.41 | 1.02 | 1.95 |
|  | Moral identity | 0.34**(0.13) | 7.22 | 1.41 | 1.10 | 1.81 |
|  | V1$\times$Moral identity | 1.12(0.31) | 13.51 | 0.92 | .56 | 1.50 |

V1 (moral superiority = 2, moral inferiority and control condition = -1)

V2 (moral superiority = 0, moral inferiority = -1, control condition = 1)

**p* <.05, ***p* <.01, ****p* <.001.
