# Supplementary material for: Self-enhancement in moral hypocrisy: Moral superiority and moral identity are about better appearances
Source: PLoS One. 2019 Jul 5;14(7):e0219382. doi: 10.1371/journal.pone.0219382 (PMC6611614; doi:10.1371/journal.pone.0219382)
Supplement: S2 Table — (DOCX) [file pone.0219382.s002.docx]

**S2 Table. Results of binary logistic regression for Choice 2 in Study 1.**

|  | | | | 95% CI for *Exp(B)* | |
| --- | --- | --- | --- | --- | --- |
|  | *B(SE)* | Wald’s $\chi^{2}$ | *Exp(B)* | Lower | Higher |
| Constant | -0.77(0.21) | 12.91 | 0.46 |  |  |
| V1 | -0.16(0.16) | 1.00 | 0.85 | 0.63 | 1.16 |
| V2 | 0.23(0.26) | 0.79 | 1.26 | 0.75 | 2.12 |
| Moral identity | 0.35(0.27) | 1.72 | 1.42 | 0.84 | 2.38 |
| V1$\times$Moral identity | -0.24(0.18) | 1.78 | 0.78 | 0.55 | 1.12 |
| V2$\times$Moral identity | 0.28(0.35) | 0.60 | 1.32 | 0.66 | 2.64 |

V1 (moral superiority = 2, moral inferiority and control condition = -1)

V2 (moral superiority = 0, moral inferiority = -1, control condition = 1)
